# Simplifying complex fault data for systems-level analysis: Earthquake geology inputs for U.S. NSHM 2023

**DOI:** 10.1038/s41597-022-01609-7

**Published:** 2022-08-18

**Authors:** Alexandra E. Hatem, Camille M. Collett, Richard W. Briggs, Ryan D. Gold, Stephen J. Angster, Edward H. Field, Peter M. Powers, Earthquake Geology Working Group, Megan Anderson, Jeri Young Ben-Horin, Timothy Dawson, Stephen DeLong, Christopher DuRoss, Jessica Thompson Jobe, Emily Kleber, Keith L. Knudsen, Richard Koehler, Daniel Koning, Zachery Lifton, Ian Madin, James Mauch, Phil Pearthree, Fred Pollitz, Katherine Scharer, Brian Sherrod, Michael Stickney, Seth Wittke, Judith Zachariasen

**Affiliations:** 1 U.S. Geological Survey, Geologic Hazards Science Center, 1711 Illinois St, Golden, CO 80401 USA; 2 U.S. Geological Survey, Earthquake Science Center, 4000 15th Ave NE, Seattle, WA 98195 USA; 3 Washington Geological Survey, 1111 Washington St SE MS 47007, Olympia, WA 98504 USA; 4 Arizona Geological Survey, 1955 E 6th St, Tucson, AZ 85721 USA; 5 California Geological Survey, 1900 S. Norfolk St., Suite 300, San Mateo, CA 94403 USA; 6 U.S. Geological Survey, Earthquake Science Center, 350 N. Akron Road, Moffett Field, CA 94035 USA; 7 Utah Geological Survey, 1594 W N Temple St, Salt Lake City, UT 84116 USA; 8 Nevada Bureau of Mines and Geology, 1664 N Virginia St, Reno, NV 89557 USA; 9 New Mexico Bureau of Mines and Geology, 801 Leroy Pl, Socorro, NM 87801 USA; 10 Idaho Geological Survey, Suite 201, 322 E. Front Street, Boise, ID 83702 USA; 11 Oregon Department of Geology and Mining Industries, 800 NE Oregon Street, Suite 965, Portland, OR 97232 USA; 12 Wyoming State Geological Survey, 1000 E University Ave, Laramie, WY 82071 USA; 13 U.S. Geological Survey, Earthquake Science Center, 525 S. Wilson Ave, Pasadena, CA 91106 USA; 14 Montana Bureau of Mines and Geology, 1300 W Park St, Butte, MT 59701 USA

**Keywords:** Natural hazards, Geology

## Abstract

As part of the U.S. National Seismic Hazard Model (NSHM) update planned for 2023, two databases were prepared to more completely represent Quaternary-active faulting across the western United States: the NSHM23 fault sections database (FSD) and earthquake geology database (EQGeoDB). In prior iterations of NSHM, fault sections were included only if a field-measurement-derived slip rate was estimated along a given fault. By expanding this inclusion criteria, we were able to assess a larger set of faults for use in NSHM23. The USGS Quaternary Fault and Fold Database served as a guide for assessing possible additions to the NSHM23 FSD. Reevaluating available data from published sources yielded an increase of fault sections from ~650 faults in NSHM18 to ~1,000 faults proposed for use in NSHM23. EQGeoDB, a companion dataset linked to NSHM23 FSD, contains geologic slip rate estimates for fault sections included in FSD. Together, these databases serve as common input data used in deformation modeling, earthquake rupture forecasting, and additional downstream uses in NSHM development.

## Abstract (summary)

**Table:** 

Measurement(s)	N/A
Technology Type(s)	N/A

## Background & Summary

Fault locations and activities are a fundamental input for traditional probabilistic seismic hazard analysis (PSHA)^1–3^. Faults are typically included in PSHA as representations of the locations where ruptures are expected to occur^4^. Deformation modeling and earthquake rupture models then combine fault geometries with slip rates, scaling relations, and magnitude-frequency distributions to form a set of synthetic ruptures^5,6^. Off-modeled fault seismicity and geodetic deformation models are also commonly included in PSHA, but the primary way that moment within the model is spatially distributed is by including fault sources and their associated slip rates^7^.

Underrepresentation of faults may lead to issues in PSHA models. Seismic sources (active faults) may be excluded due to inclusion criteria for a given model (such as geologic slip rates), or perhaps excluded because a given active fault has yet to be identified^8^. As a result, seismic hazard calculated with a minimum fault model containing only a subset of known Quaternary active faults might be poorly estimated in space. A subtler issue is that contributions from off-fault seismicity and geodetic deformation models may be too high without more faults to distribute the moment contributions from fault-based deformation models^9^. The inclusion of off-fault seismicity and off-fault moment from geodetic models is a critical component of PSHA because the off-fault sources capture information missing from the modeled fault system. A related approach, which we leverage here, is to provide the most complete Quaternary-active fault network possible, moving toward the option of a maximum fault model that includes as many known Quaternary active faults as possible. Often, numerous faults are mapped and known to be active but are not incorporated into seismic hazard analyses due to inclusion criteria that excludes faults with a lack of geologic slip rate studies^10^. The inclusion of more faults, even at low rates of activity, provides a more complete representation of on-fault moment rate^11^. Large-scale contributions toward seismic hazard as measured by, for example, total moment rate within the geologic deformation model, may not be greatly affected by adding more low-rate faults. However, small-scale/site-based calculations may be influenced by representing more known faults in PSHA^12^. Inclusion of a more complete fault inventory also allows rupture to propagate more realistically along fault networks.

## Previous Work and Current Update

**Fig. 1:**
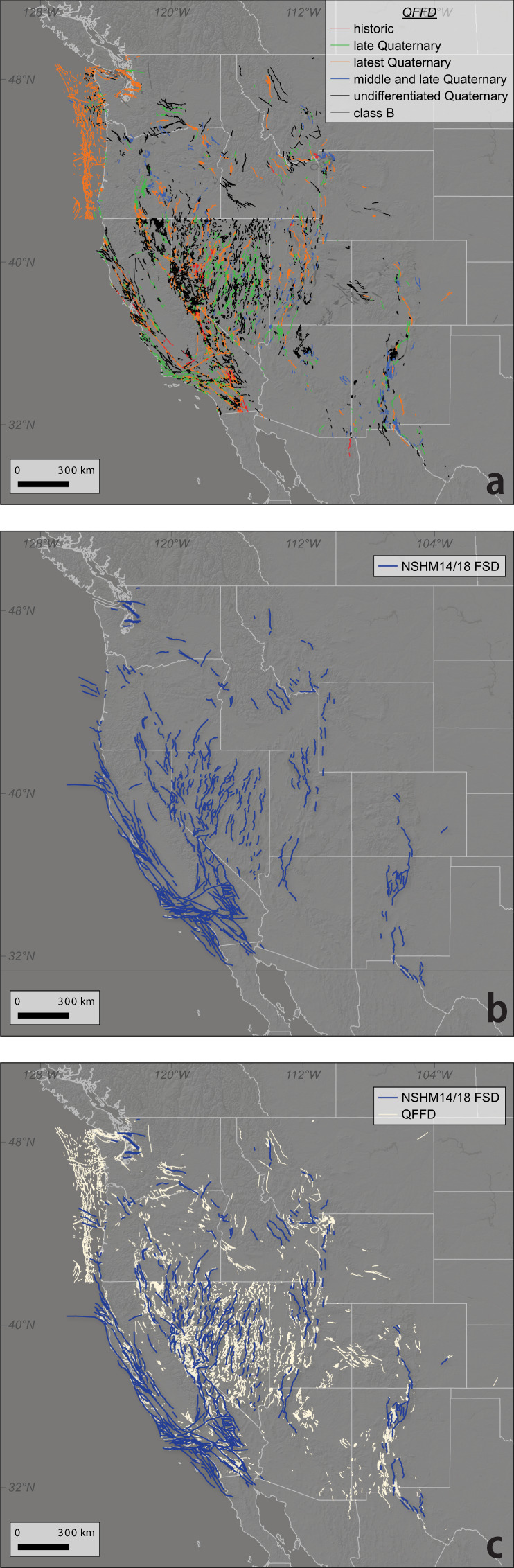
Fault databases available across western U.S. prior to NSHM23 update. (**a**) USGS Quaternary Fault and Fold Database (QFFD) colored by recency of activity category. (**b**) NSHM14/18 fault sections database (FSD). (**c**) Overlay of NSHM fault sections on QFFD (single color for clarity) to highlight spatial mismatch between databases.

It has been common practice to exclude faults from seismic hazard models for which field-derived slip rates are not available. For example, in prior iterations of the U.S. National Seismic Hazard Model (NSHM), fault sections were only considered if there was a slip rate derived from field measurements along the fault^1^. This approach is reasonable because practitioners have excluded faults with only approximated slip rates. However, such inclusion criteria significantly limited the number of active faults in the 2014 and 2018 NSHM versions^13^. No updates were made to the fault sections database between the 2014 and 2018 NSHM releases. Herein, the faults considered by NSHM14/18 will be referred to as NSHM18 FSD (fault section database). NSHM18 FSD considered ~650 fault sections for hazard modeling across the western United States (U.S.). In contrast, more than 2,000 faults are known to be active during the Quaternary (since 1.8 Ma^14^) across the western United States^15^. (Fig. 1). The U.S. Geological Survey (USGS) Quaternary Fault and Fold Database (QFFD), which is regularly updated by individual state geological surveys, provides the most comrehensive view of Quaternary-active faulting in the western U.S.^16^. Many of the faults included in QFFD are characterized as Quaternary active due to stratigraphic, geomorphic, and geochronologic relationships. However, most of these faults have not yet been studied in detail to determine site-specific geologic slip rates.

For the State of California, the mismatch between known Quaternary-active faults and modeled fault sections in seismic hazard analysis was addressed during the update from version 2 to version 3 of the Uniform California Earthquake Rupture Forecast (UCERF)^17^. This update resulted in an increase from ~200 faults to ~350 faults. The UCERF3 update used fault representations from regional databases (e.g., Southern California Fault Model; SCFM) and assigned ranges of allowable slip rates for faults lacking a field-derived slip rate^10^. The purpose of including more faults in the UCERF3 update was two-fold: (1) to incorporate the fullest possible range of known active faults into the hazard calculations, and (2) to better reflect observed connectivity between faults and fault systems, allowing for the possibility of multi-fault ruptures^6^.

To address the mismatch between mapped and modeled faults in the western United States, we utilize information compiled in the QFFD. The QFFD contains the most up-to-date and far-reaching compilation of Quaternary faulting in the western United States. Here, we define the western United States as the states of Arizona, California, Colorado, Idaho, Montana, Nevada, New Mexico, Oregon, Texas, Utah, Washington, and Wyoming. Several barriers exist to simply importing QFFD fault data into the next update of the NSHM. The primary barrier, noted above, is the lack of field-derived slip rates for most faults. Another fundamental issue is that the geometric representations of faults in QFFD represent the expression of faulting recorded at the Earth’s surface, but such complex surface traces are not immediately suitable for inclusion in the hazard model. This is because fault sources used in seismic hazard modeling represent the fault plane at seismogenic depths^2,4,18,19^. Although we do not know exactly what the fault looks like at seismogenic depths, a common assumption is that faults simplify with depth^20^. As such, the geometric representations of faults in PSHA are typically relatively coarse and generalized for the purpose of calculations. Faults in PSHA are also often redrafted and simplified to enforce geometric consistency across faults with varying degrees of study. Geometric fault simplification of fault sources is common practice in the construction of seismic hazard models. For example, faults were simplified as a part of the UCERF2 to UCERF3 update where needed, as well as in compilations of other fault section databases, such as the Fault2SHA database across the Italian Apennines^17,18^.

In most active fault databases, geologic slip rates are linked directly to fault sections and recorded as an attribute in the geometric database, similar to dip degree and rake^21^. This approach effectively requires that a fault section or ‘segment’ have a single slip rate, often reported without uncertainty. Assigning slip rates to entire fault sections can lead to a loss of important information, such as the precise location of the slip rate study location (including uncertainties surrounding that particular location along the fault and the slip rate site itself), and the parameters used in the calculation of the geologic slip rates^6,10^. In the UCERF3 update process, a slip rate database that preserved site-specific information and that was not explicitly linked to fault geometries was created and implemented in the deformation modeling workflow. This compilation (UCERF3 Appendix B) documented the location, slip rate values, range of permissible slip rate values, and various interpretative details such as time frame over which the slip rate was calculated^10^. UCERF3 Appendix B has proven instrumental in research applications, such as the comparison between geodetic and geologic slip rates^22–24^, comparison of maximum rupture models for plate boundary faults to slip rates along strike^25^, and as a benchmark in numerical modeling studies^26^. For seismic hazard analysis, the decoupling of slip rate data from fault geometry was important for guiding and benchmarking multiple geodetic deformation models constructed in UCERF3^27^. Point-based geologic slip rates provide geodetic deformation modelers with the possibility of resolving slip rate variability along a fault. Additionally, these data can be further considered when tackling rupture segmentation and the application of fractional slip rates at sub-fault section boundaries^28^.

In this paper, we address the general problem of creating a fault section database and curating fault slip rate data for seismic hazard analysis. We demonstrate our approach with specific examples across the western United States from the planned 2023 update to the NSHM. Our approach for building a fault section database (FSD) includes reviewing previously defined faults, incorporating new faults from published sources, and simplifying fault traces using a uniform treatment. We create a companion database of slip rates (EQGeoDB) that includes site-specific studies and at least one estimated slip rate for each fault section presented with author-reported uncertainties. The database of geologic slip rates, coupled with the updated fault sections, provides a complete set of input data that can be distributed and implemented in the internal NSHM workflow and in subsequent PSHA models. The databases, including a reference list and a log of all changes made to the fault sections database, are freely available as a USGS data release at 10.5066/P9AU713N^29^. Although these databases were generated for use in the NSHM23 update, the databases are designed such that geologists, geodesists, and other Earth scientists may implement these data in further research applications.

## Data Summary

**Fig. 2:**
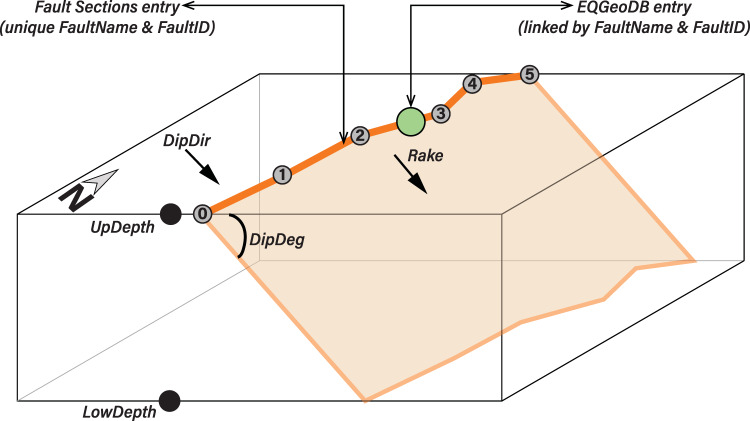
Schematic diagram of hypothetical fault in plane and cross-section view. Numbered gray circles represent the ordering of coordinates in list form to uphold right-hand rule convention in the fault sections database. The larger green circle represents the location of an EQGeoDB entry. Fault sections attributes highlighted here are further described in the Database Fields section.

The NSHM23 fault sections database (NSHM23 FSD) and earthquake geologic slip rates database (EQGeoDB) are two separate, yet linked, databases (Fig. 2). The fault sections database consists of line features, whereas EQGeoDB consists of point features, which are linked through common FaultID numbers.

The NSHM23 FSD contains a total of 1,017 fault sections, with 8 of those included as proxy faults to represent broad zones of distributed deformation. In addition, nearly 500 slip rate study sites were compiled in the EQGeoDB across the western United States, which builds from the nearly 250 entries from UCERF3, Appendix B^10^. The data span the western United States, including the States of Arizona, California, Colorado, Idaho, Montana, Nevada, New Mexico, Oregon, Texas, Utah, Washington, and Wyoming. Updates pertaining to the central and eastern US, as well as Alaska, are topics of separate but related efforts for the NSHM23 update^30,31^. At this time, no fault sections database for use in probabilistic seismic hazard analyses exists for Hawaii.

Overall, the NSHM23 FSD (n = 1,017 faults) represents a 58% increase in the number of fault sections compared to NSHM18 FSD (n = 646 faults). The Intermountain West region (Arizona, Colorado, Idaho, Montana, Nevada, New Mexico, Texas, Utah, and Wyoming) had the largest increase, with a 138% increase in fault sections. Washington also had a two-fold increase in fault sections due to the recent update of QFFD in that region^32^. California had the fewest additions to the fault sections database, as this update process had previously occurred in the transition from UCERF2 to UCERF3.

**Fig. 3:**
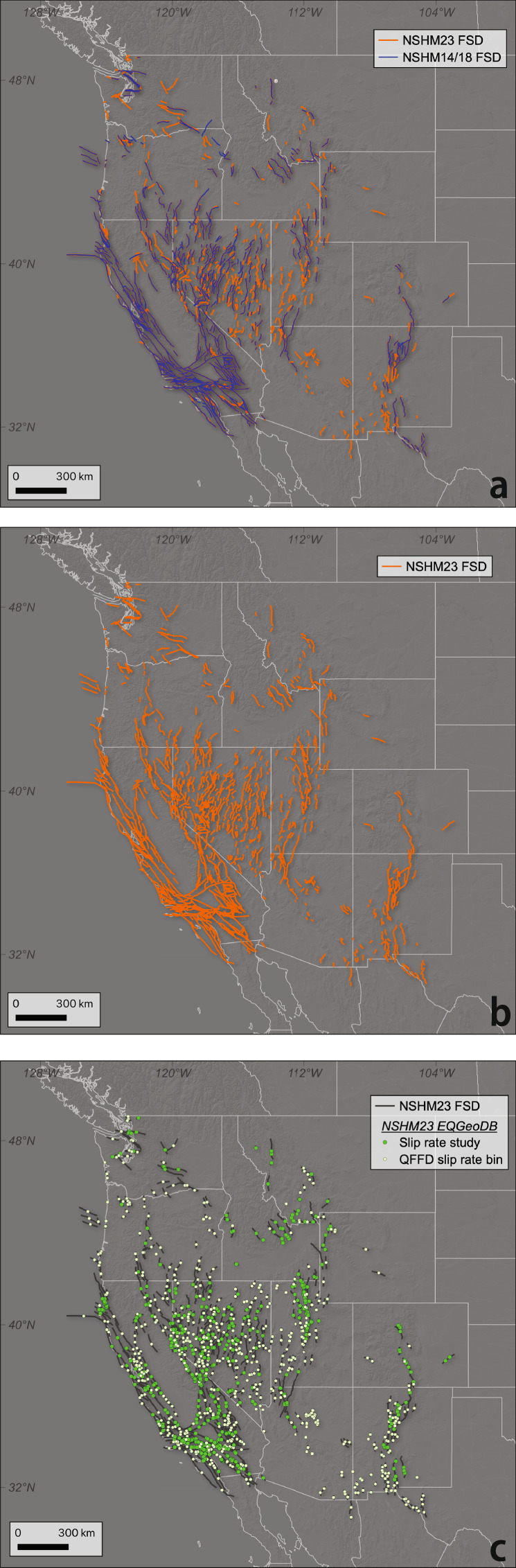
Updated databases across the western U.S. (**a**) Overlay of NSHM14/18 fault sections on NSHM23 fault sections to highlight spatial distribution of additions to the database. (**b**) NSHM23 fault sections. (**c**) Overlay of EQGeoDB on NSHM23 fault sections. Bright green circles indicate where studies have been completed or where rates have been assessed by the community. Light green circles indicate fault centroids where QFFD slip rate bins are recorded for use in deformation modeling.

**Fig. 4:**
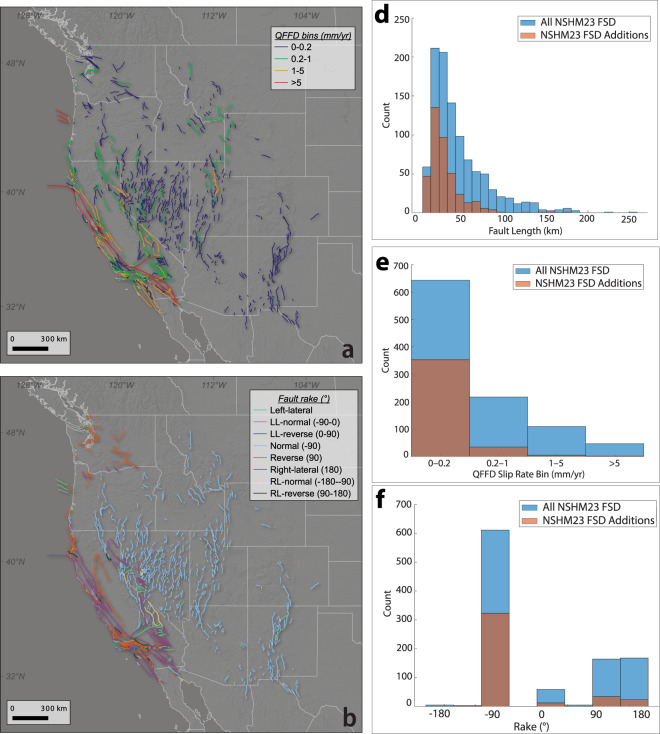
Maps of NHSM23 fault sections database colored by (**a**) QFFD slip rate bin and (**b**) style of faulting. RL: right-lateral; LL: left-lateral. Histograms showing distribution of fault length (**d**), QFFD slip rate bin (**e**), and rake (**f**) for all NSHM23 fault sections and NSHM23 fault section additions only.

The augmented NSHM23 FSD contains shorter, slowly slipping normal faults across the western United States compared to the existing NSHM18 fault sections database (Figs. 3 and 4). The average fault length of added faults is 27 km, compared to the average fault length of 43 km across the entire NSHM23 FSD. Likewise, ~90% of the added faults fall within the 0–0.2 mm/yr QFFD slip rate category. In comparison, ~60% of all faults in the database fall within that same category. Finally, 80% of the newly added fault sections have a rake of ~-90°, compared to 60% across the entire NSHM23 fault sections database. These comparisons highlight that NSHM18 FSD covered primarily the longer and faster faults across the western United States, and the NSHM23 FSD update adds many low strain rate, long recurrence interval (slip rate <0.2 mm/yr) normal faults to the database.

Although most (>80%) of the newly added fault sections do not have prior studies of geologic slip rates, the EQGeoDB slip rate compilation incorporates ~280 slip rate study locations across the western United States that were considered in NSHM18 FSD. In addition to these previously identified sites, we add ~40 sites included in the EQGeoDB from newly considered faults. Data compiled up to c. 2013 in UCERF3 Appendix B^10^ are included in EQGeoDB as originally listed in that database. In addition to the nearly 250 entries from UCERF3 Appendix B, our current effort resulted in the addition of ~15 new sites included from California that have been published since UCERF3.

In our approach, the EQGeoDB represents data mined from publications in a tabular form, whereas the NSHM23 FSD is a more interpretative database which required many layers of expert review and assessment despite the implementation of automated simplification techniques. Fault mapping is inherently a scale-dependent process, making fault geometries an inherently scale-dependent product, and we attempt to standardize the geometries in an internally consistent manner. As such, we ingest published information to reinterpret each fault section geometry. In contrast, the EQGeoDB is a collation of published information. Minimal to no additional interpretation is applied to author-reported data entered into EQGeoDB. The purpose of this is two**-**fold: (1) to accurately represent field-derived data as a priori information to constrain geologic and geodetic deformation models, and (2) to provide a record of the available literature. This style of data compilation follows the foundation put forth in the creation of UCERF3 Appendix B^10^.

## Methods

The NSHM23 FSD and EQGeoDB were compiled following a review of the prior fault sections used in the 2014 and 2018 versions of the NSHM (NSHM18 FSD^21^), literature review of peer-reviewed, publicly available publications (accepted as of December 2020^33^), geometric simplification of all faults included in the QFFD for the FSD, and subsequent collaborative iterations in the form of public workshops that included state and federal partners and stakeholders in each state or region.

Our goal was to decouple the fault geometries from the geologic slip rates to have two complete, independent but related databases. This allows for the use of site-specific slip rates in deformation models, along with the inclusion of numerous metadata fields for each rate, as was done in UCERF3 Appendix B.

### Fault sections database (FSD)

To maintain consistency across this update, we reviewed the NSHM18 FSD geometries and associated parameters before considering the addition of fault sections to the NSHM23 FSD. The NSHM18 FSD contains both fault geometry and parameters, such as rake and lower seismogenic depth, as well as activity (slip rate) following weighting of individual slip rates from different deformation models. The review of the existing fault sections database led to the creation of inclusion criteria for potential additions to the database. The criteria were:

1. Definitive evidence of Quaternary tectonic deformation.
2. Fault length must exceed 7 km.
3. Evidence of faulting and associated geometry must be available in a peer-reviewed, publicly available publication.

Some faults that were previously included in the NSHM18 FSD did not necessarily meet all the above criteria, particularly the third item. Some fault geometries have been carried through iterations of NSHM based upon unpublished consulting or technical reports, conference abstracts and field trip guides. Although NSHM18 FSD included references from “gray literature” that are not generally available and are not always peer-reviewed, we limit the scope of new information to peer-reviewed, publicly available publications (including journal articles, map publications, and state geological survey reports). We opted to include those legacy representations based on gray literature into the updated database but did not include new additions to the fault sections database unless the above criteria were met. References for the basis of updating the fault sections database are available in the FSD Data Repository under “Change Log^29,34^”, which documents the changes between NSHM14/18 FSD and NSHM23 FSD. The references used to make any changes or introduce new faults into the database are primarily based on the QFFD legacy reports and references therein. The legacy reports within QFFD are available through the web database search tool^35^. In some cases, publications that post-date the QFFD reports (typically prepared in the 1990’s and last updated in c. 2013) were utilized.

Although many faults from NSHM18 FSD were carried over to NSHM23 FSD with no changes (n = 417), some fault sections were updated to reflect a more realistic geometry (n = 160). Such updates reflect recent (post c. 2013) or previously unconsidered publications. Additionally, some faults that were included in NSHM18 FSD were not included in NSHM23 FSD (n = 69). Most faults subtracted from the NSHM18 FSD represent the removal of alternative fault representations used in California. Unlike UCERF3, no alternative fault model is planned for this update. Should the need and demand to incorporate alternative fault models arise, multiple fault representations can be supported in future iterations of FSD. UCERF3 alternative fault representations can be found in NSHM18 FSD^21^. When selecting between alternative faults from UCERF3 to carry on to NSHM23 FSD, we favored fault representations that enabled more connectivity in the fault model following precedent set in UCERF3^6,17^. A few faults (n = 25) were excluded from NSHM23 FSD due to a lack of unequivocal tectonic deformation during the Quaternary.

#### Geometric simplification of QFFD for use in NSHM fault sections

Detailed fault mapping completed by field geologists, while representative of surface observations, may not be representative of fault geometry at depth^36^. Although a short fault strand may be observed at the surface as part of the local fault zone, each fault trace mapped at the surface does not represent an individual source capable of a seismic rupture ~M6.5, which was the minimum threshold for on-fault ruptures in prior iterations of the NSHM. More likely, short, discontinuous traces merge at depth and/or rupture in conjunction with a deeper, simpler trace. Given that the NSHM23 FSD was designed for use in PSHA, which is concerned with probability of shaking at any location, the discretization of faults is expected to represent the surface that causes the shaking, not the displacement. If these fault sections were intended for use in probabilistic fault displacement hazard (PFDHA), which is concerned with the amount of ground displacement at any given location, such detailed knowledge of the location, number, and distribution of faults across a fault zone would be required^37–39^. Given the intended use case of NSHM23 FSD in PSHA, we focus on the simpler representation of any given fault while providing minimal, long wavelength geometric realism to the surface fault traces (e.g., following topographic/geomorphic fault traces).

**Fig. 5:**
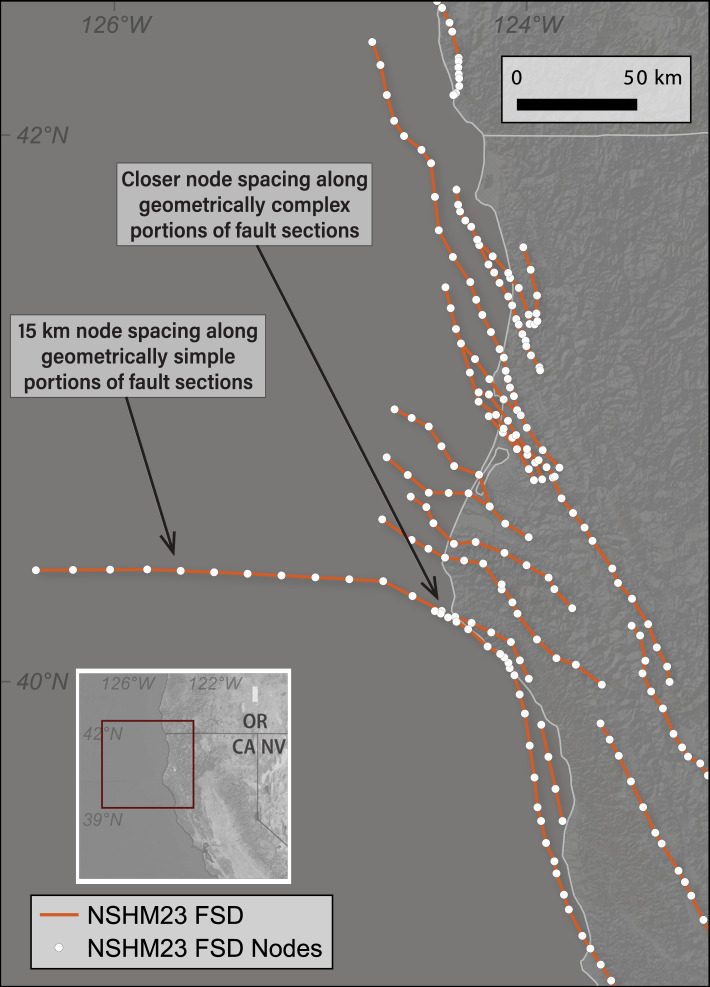
NSHM23 FSD (orange lines) and node locations (white circles) for coastal northern California/southern Oregon. Inset shows relative location. NSHM constraints specify that no distance between nodes along a fault section should exceed 15 km.

Because detailed fault mapping, such as the fault representations within the QFFD, are intended for use by the geologic community, and not for seismic hazard modeling, this simplification step is essential to ensure common, generalized representations of all faults. Additionally, given that the QFFD receives contributions from multiple sources and contains information submitted over the past ~20+ years, there are differences in representation styles and resolution of different faults included. The goal of the simplification step is to have a minimum node spacing along a given fault section of ~1 km, following node discretization set by NSHM18 and UCERF3^13^. An additional node spacing prerequisite was to set a maximum node spacing of 15 km (Fig. 5). In this example of faults along the northern California coast, we see that some nodes are arbitrarily added to straight portions of long faults, such as the Mendocino fault section, whereas other more geometrically intricate faults require more nodes and approach the 1-km node spacing. Additionally, the minimum fault length of an individual fault section is set at 7 km because ruptures shorter than 7 km are unlikely to have a magnitude >6.5. Finally, we ensure that, following simplification, faults were drawn in the direction that honors the right-hand rule convention (that is, a fault dips to the right-hand side when looking in strike/draw direction)^40^ (Fig. 2).

The geometric simplification of the QFFD faults was first completed algorithmically, and then it was validated and merged by human users to ensure that the simplification was reasonable given geomorphic and topographic context. These steps were completed in a standard geographical information system (GIS) environment. The QFFD was last accessed in May 2020 for the simplification of line features. QFFD is publicly available to view and download^14^.

The smoothing process steps are as follows:

1. Snap very closely spaced nodes together (50–100 m).
2. Smooth fault segments within a buffered tolerance of 300 m.
3. Group and manually merge similar fault sections based on fault attributes.
4. Verify node spacing (~1 km), fault length (≥7 km), and right-hand rule.

The first step, snapping very closely spaced nodes together, smooths over inadvertent gaps in the geospatial representation of a given fault. Because the line work is submitted and compiled by many different mappers, some faults are represented by many discontinuous fault strands while others are continuous line features. This first step provides a connected fault that is smoothed based on a defined buffer azimuth in the second step. The smoothing algorithm reduces unnecessary and unrealistic deviations from the detailed mapping. These first two steps are completed by grouping faults under the attribute of fault name; sub-sections of a given fault may be defined by different names in the QFFD depending on the original compiler.

After these first two steps are completed algorithmically, each simplified fault section is considered by human review in map view to determine where and how fault sections may be merged. For example, a gap distance between simplified fault strands of ~50–100 m will be merged, under the rationale that, while that gap in fault trace may be observed at the surface, the gap likely does not persist to seismogenic depths. But, if a gap between fault sections on the order of kilometers persists (>5 km^6^), this gap is retained, and two fault sections are broken out. Fault sections are considered for merging based on their attributes recorded in the QFFD archived reports. In addition to the fault geometry, each line feature in QFFD has 18 fields with a fault-specific reference list in its current formulation (legacy reports of QFFD contain more information and text-based descriptions, but such reports are no longer supported).

We utilized QFFD attributes such as dip direction, sense of movement, and most recent prehistoric deformation when merging fault sections. For example, if the southern portion of a given fault dips to the west, and the northern portion dips to the east, and these dip directions were persistent along the two subsections of fault, such a hypothetical north-south trending fault was subdivided based on this difference in dip direction. Furthermore, if portions of a fault were categorized as having different bins of fault activity, whether slip rate or the recency of activity category in QFFD, these faults were separated into different sections under the assumption that they may rupture independent of each other. Once fault representations were merged and simplified, their geometries were verified to contain only a single line segment per unique fault ID and fault name, have reasonable node spacing, be greater than 7 km in length, and drawn in the direction to abide by right-hand rule convention.

**Fig. 6:**
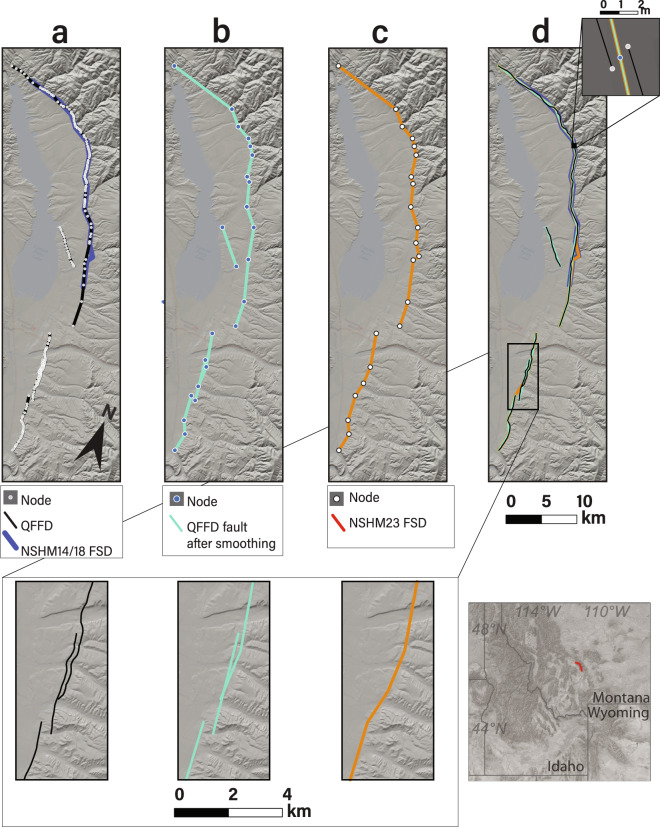
Example of QFFD geometry simplification from Canyon Ferry fault (Montana), with QFFD fault geometry prior to smoothing (column a), QFFD after smoothing (column b), NSHM23 FSD (column c), and all three representations plotted together (column d). Panel E shows very small distances between ends of line segments in QFFD. Insets in the bottom row of columns **a**–**c** show zoomed in view of Canyon Ferry (Totson) section. Inset in lower right corner of figure shows general location of Canyon Ferry fault system.

Figure 6 highlights an example of the simplification process from the Canyon Ferry fault in Montana. The QFFD representation of the Canyon Ferry fault has 218 nodes spread over 6 individual fault traces spanning a total length of ~84 km with an average of 3 nodes per kilometer. Additionally, the southern section of the Canyon Ferry fault, although likely intended to be a single section, is truncated and separated by <2 m into two discrete fault sections, which is likely a result of digitization error. Not only is this gap inadvertent, but such a short discontinuity at the surface likely does not provide meaning for the fault geometry at seismogenic depths. Additionally, the snapping algorithms smoothed and connected these meter-scale steps along what is expected to be a continuous fault trace. After completing the above workflow, the number of nodes were reduced from 218 to 22, a decrease by roughly a factor of 10. Based on the mapping of the Canyon Ferry fault within QFFD, including the 5-km gap between the southern terminus of the southern section and the northern end of the Totson section, we opt to include two separate Canyon Ferry fault sections.

#### Proxy faults

**Fig. 7:**
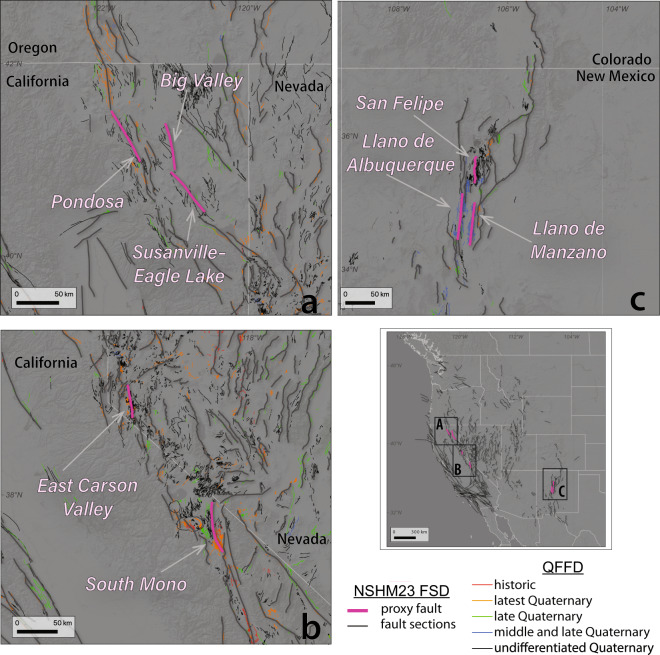
Maps showing locations of proxy faults (pink) across northeast California (**a**), the Reno-Tahoe area (**b**) and the Rio Grande Rift (**c**) in comparison to other NSHM23 fault sections (gray) and the regional QFFD (multi-colored by recency).

Where faults could not be reasonably simplified given a lack of confidence for how a single fault accommodates broad zones of distributed deformation (1–10+ km wide), a geometrically simple proxy fault provides representation in the fault sections database (Fig. 7). Here, the definition of the main fault trace within a broad zone of surficial scarps necessitated further simplification of the faults system onto a truly idealized fault trace. In later steps of the PSHA workflow, the strain collapsed onto the proxy faults in deformation modeling will be redistributed into an areal source (similar to C-Zones used in UCERF2 and NSHM2008^41^). We included eight proxy faults in NSHM23 FSD. These proxy faults were delineated in northeast California, west-central Nevada, and the Rio Grande Rift (Fig. 7). The definition of the polygons about these proxy faults occurs in subsequent steps within the NSHM workflow.

#### Fault segmentation

**Fig. 8:**
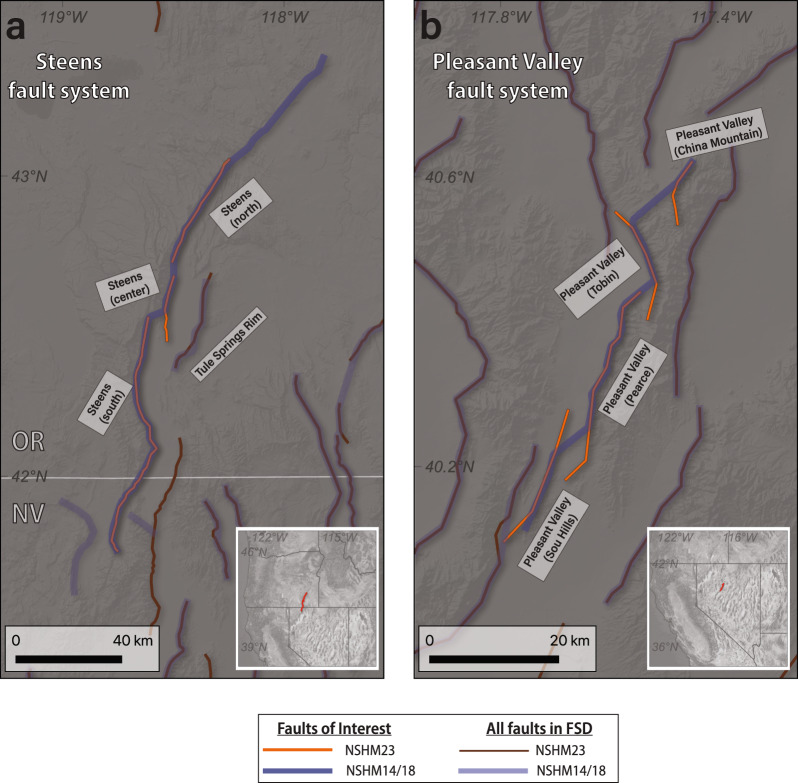
Comparison of segmentation of NSHM14/18 FSD single fault section into multiple fault sections in NSHM23 FSD. Example shown here from Steens (**a**) and Pleasant Valley (**b**) faults systems (inset shows general location of faults).

In addition to fault simplification, and to enable the possibility of applying multi-fault rupture simulations (e.g., UCERF methodology) to small regions within the western United States, some fault sections included as single faults in NSHM18 FSD are now segmented – meaning the faults are separated into more fault sections – in NSHM23 FSD. While the application of a UCERF-type inversion approach has only been applied in California and the Wasatch Front^42^ to date, providing a segmented fault sections database, such as NSHM23 FSD, provides some flexibility in potentially applying the inversion over small regions of interconnected faulting elsewhere. The segmentation decisions arose primarily from the QFFD fault trace simplifications (e.g., relating QFFD attributes to NSHM23 FSD geometries) and expert interpretation of those results. The Steens (Oregon) and Pleasant Valley (Nevada) faults were represented as single fault sections in NSHM18 FSD but are now represented with numerous fault sections in NSHM23 FSD (Fig. 8). In NSHM23 FSD, we represent Steens as a system of three fault sections and Pleasant Valley as a system of four fault sections. The goal of such separation of faults, in the case of the Steens fault example, allows for both shorter ruptures with small to moderate magnitudes along the previously very long Steens fault section (~300 km long), as well as the possibility of multi-fault ruptures across portions of its own subsections and the nearby Tule Springs Rim fault (should a UCERF-style inversion be applied to this region). Additionally, Fig. 8a highlights the shortening of the northern extent of the Steens fault; the fault length here is truncated due to a lack of unequivocal Quaternary tectonic deformation.

### Geologic slip rate compilation (EQGeoDB)

#### EQGeoDB definition and purpose

In tandem with NSHM23 FSD, the companion geologic slip rate database (EQGeoDB) was prepared. The EQGeoDB contains geologic slip rate data and metadata. Geologic slip rate information is commonly considered with geodetic data to develop deformation models^7,27^. The geologic slip rates included in EQGeoDB represent potential a priori constraints for use in the development of deformation models for NSHM23. A given geodetic deformation model may choose to severely limit the geodetic results to the geologic rates, loosely constrain the range of rates, or only return to the geologic rates as a benchmarking exercise after an initial model run. All approaches of geodetic deformation modeling are supported by the EQGeoDB.

Outside of the NSHM23 application, EQGeoDB may be used by practitioners to understand the distribution of geologic slip rate data in a given field area, benchmark numerical modeling studies, or complete large-scale, regional analyses^11,43,44^.

#### EQGeoDB compilation

The main sources of data used to compile the EQGeoDB were primarily from the documentation for slip rates used in NSHM18 and UCERF3, as well as the QFFD archived reports and the references therein. The text-based descriptions of slip rates recorded from NSHM14^45^ were transposed into numerous fields, which describe the offset feature, the geochronologic determination of the offset feature, and general observations made at each site. Additionally, a location was assigned for each entry in EQGeoDB. While these locations were compiled for California faults^10^, the locations were not recorded for sites outside of California in past efforts. As such, in this current effort, the location of these sites was determined from the original sources and maps included therein. In the rare case that the original location of the slip rate study did not fall precisely on a simplified fault geometry, we assigned a location as close as possible to the study site. Finally, a literature search of slip rates across the western United States published from c. 2013 onward was conducted to include the best available data in the EQGeoDB. This resulted in the addition of ~15 new sites within California that post-date UCERF3 Appendix B, in addition to the inclusion of ~250 sites outside of California across the Intermountain West and Pacific Northwest regions. As with the fault sections database, information used in previous NSHMs that did not meet the new criteria for inclusion regarding publication status was “grandfathered” into the NSHM23 databases; only peer-reviewed and publicly available publications were included in the EQGeoDB for new information introduced to the NSHM workflow.

Given that most faults newly considered in NSHM23 FSD do not have site-based or otherwise investigated observations of slip rate along their length, we utilize the QFFD slip rate categories. The slip rate categories are: < 0.2 mm/yr, 0.2–1 mm/yr, 1–5 mm/yr, and > 5 mm/yr. We truncate the slowest bin at 0 mm/yr (no negative slip rates are allowed) and limit the fastest bin at 35 mm/yr (approximately the slip rate of the fastest faults considered in NSHM23 FSD). Because these bins do not apply to a specific location, we include them spatially in EQGeoDB at the approximate centroid of a given fault section. Additionally, we include rates that are estimated by different means, such as regional comparisons of basal facet heights and other geomorphic relationships^46,47^ and consensus rates used in other regional hazard assessments^48–50^. These rates are also inherently not site-specific and are therefore applied at the approximate centroid of a fault section. These rates are flagged as such and are not considered as a “slip rate study” (see section “Database Fields” for further discussion on these flags).

Although most common in California, numerous faults across the western United States have multiple estimates of geologic slip rate along a single parent section. For example, the Lemhi fault (Idaho) has seven sites along its length with estimates of slip rates. Here (and at many sites along other Basin and Range faults), these sites consist of observations of tectonic displacement (vertical separation of a surface across a fault scarp) and a measured or estimated age of the surface offset or otherwise constraining the timing of the vertical separation. Some such observations along the Lemhi fault, and many others across the western U.S., come from trench (exposed) stratigraphy or surficial observations.

Numerous slip rate locations across the slowly slipping faults across the western United States record relatively few (commonly one or two) earthquakes. Although these few-event records may not be indicative of the long-term fault behavior^51^, these geologic slip rates are still compiled in EQGeoDB. With ample metadata collected for each slip rate (such as number of events averaged over and size of offset), each geologic slip rate estimate contains information for an expert user to assess the uncertainty inherent in each rate calculation. Although geologic slip rate uncertainty is included in EQGeoDB where such information was available, we did not perform a uniform treatment of slip rate uncertainty throughout the database. Rather, the values of EQGeoDB represent the reported rates or offset/age values from a given author.

Within EQGeoDB, a field called “ReptReint” records whether the original authors have reported the rate as it is recorded in the table (“Rept” = reported) or if the rate is calculated from offset and age observations listed by the authors without calculation of a rate (“Reint” = reinterpreted). In some cases, the original data source for geologic slip rate used in NSHM18 FSD was reinterpreted, yielding a slightly different geologic slip rate value; the result of this change was typically a reduction in geologic slip rate on the order of ~10–20%. The practices described here follow the precedent set by UCERF3 Appendix B slip rate compilation.

### Regional expert verification

Drawing on regional expertise was critical to the development of the NSHM23 fault sections database. Unlike the UCERF3 update, this NSHM23 update spans the entire United States In this present effort, we focused on the 12 western states (California, Washington, Oregon, Idaho, Nevada, Arizona, New Mexico, Utah, Montana, Wyoming, Colorado, and Texas); the faults of the central and eastern U.S. and Alaska are considered separately from the western United States faults^30,31^. This large geographic and tectonically diverse region deserves special local attention to the faults in each sub-region of Pacific Northwest, Intermountain West, and California. To this end, we presented preliminary drafts of the simplified fault networks described in the previous section from each state to the local experts (typically state geological surveys). We then worked iteratively to ensure that local knowledge, both the fault geometry and attributes, as well as the presence or absence of unequivocal tectonic deformation in the Quaternary, was represented in NSHM23 FSD. This partnership with state colleagues enabled validation of the QFFD legacy reports (which have not been updated since c. 2013 as they are no longer maintained). As a result, we were directed to newer literature and geologic maps that provided alternative and complementary representations of faults, which are reflected in the NSHM23 FSD.

### Public feedback

Following the iteration and refinement of the provisional fault sections database with state partners, three virtual regional workshops were held in November 2020 to present draft results of our work. The workshops, which focused on Intermountain West, Pacific Northwest, and California regions, saw participation from state and federal experts, consultants, and academics with nearly 300 participants across the workshops. The workshops provided an opportunity for the public to comment on both our process and draft results. Following the workshops, a period of open discussion and review ensued, with more than 50 workshop participants providing written feedback that was incorporated into the databases.

### Limitations of the datasets

Although the updates to NSHM23 FSD and EQGeoDB enable improvements in both seismic hazard analyses and future research directions, the databases have some key limitations. For example, the FSD is derived in large part from the long-existing QFFD, which has a complex and patchwork history. In detail, the QFFD database synthesizes contributions from a large number of individuals and organizations with heterogeneity in mapping and attribution styles. Thus, the QFFD represents our best but, at times, inconsistent knowledge of active faulting. Additionally, simplification of faults was intended to best characterize seismic sources at depth, which differs from the practical use case of QFFD. As modeling techniques move toward inversions that propagate complex rupture along three-dimensional fault networks (e.g., UCERF3) or physics-based approaches (e.g., RSQSim^52,53^), details of the subsurface structure and interconnectedness will become more important^2^. Databases such as Fault2SHA retain multiple representations of a given fault, which allows for one database to provide cohesive, internally consistent representations of the same fault structure^18^. However, the relatively small regional scope of the Apennines (Fault2SHA^18^) (~40 fault sections) compared to the much larger western United States region (NSHM23 FSD) (~1,000 fault sections) precluded such work at this time. Finally, faults that have ruptured historically typically present a greater depth of detail in fault mapping and can potentially obscure the differentiation between observations of the most recent earthquake versus the long-term signal of rupture of a fault section at depth. For example, the Pleasant Valley fault system (Fig. 8b) has been studied in extensive detail following the 1915 Pleasant Valley earthquake. The presence of historical ruptures, including the 2019 Ridgecrest, 1999 Hector Mine, 1992 Landers, 1983 Borah Peak, 1959 Hebgen Lake, 1954 Rainbow Mountain, and 1932 Cedar Mountain ruptures (all of which have associated causative faults represented in NSHM23 FSD), potentially present a “spotlight” issue, shining a more detailed light on faults with recent, observable surface deformation. Surface rupture mapping of coseismic deformation immediately after an earthquake^54^, retrospective geomorphic mapping of potentially causative fault features in the geomorphology^55^, or mapping 3D planes based on aftershock relocation^56^ well represent the fine-scale fault structure, but are too detailed for 1:1 inclusion into the FSD.

Limitations also exist within the EQGeoDB. Although the decoupling of geologic slip rates from fault sections provides an opportunity for analysis of fault behavior and rupture patterns, the inclusion of numerous slip rate estimates at different locations along a fault also presents some challenges. Notably, the slip rate estimates themselves may or may not be internally consistent, either along fault strike or over the Quaternary history of a fault. By including more data, the rates may require reconciliation to arrive at a reasonable along strike rate. Most importantly, the EQGeoDB does not account for the number of earthquake cycles over which a given slip rate was averaged. Theoretical and numerical modeling studies indicate that average slip rates over short intervals do not record the long-term behavior of a given fault^51^, but this was not accounted or corrected for in EQGeoDB. Furthermore, treatment of geologic slip rate uncertainty across slip rate studies is not uniform; this large undertaking is a topic for future development. Finally, the current version of EQGeoDB (version 2) only includes geologic slip rate data, which are only one part of the earthquake geology data that can be used to describe a fault. Although some information is recorded in the metadata for a given rate, no entries in EQGeoDB directly describe paleoseismic or slip per event histories of a fault. Such augmentation of EQGeoDB to supplement the geologic slip rate data already included with paleoearthquake chronologies and along fault coseismic displacements is planned. Given the NSHM workflow schedule, the geologic slip rate data collection was prioritized over additional datasets for the current database release.

## Data Records

The NSHM23 FSD and EQGeoDB are available as a U.S. Geological Survey data release at 10.5066/P9AU713N via ScienceBase^29^. A Community Page has been established on ScienceBase at https://www.sciencebase.gov/catalog/item/5fe1149ad34e30b9123f0160 where all earthquake geology input and output data will be stored for use in the NSHM23^57^. This community page will also include the fault sections database from Alaska and the central and eastern U.S. as such databases become available. The Community Page is planned to house both the most up-to-date, as well as deprecated, databases that have been refined in the NSHM23 update process.

### File formats

The fault sections database was generated in a GIS framework and was manipulated in the ESRI Shapefile (.shp) format. Additional file formats are prepared and provided, as comma separated value (.csv), Keyhole Markup Language (.kml), and geoJSON (.geojson) formats for ease of use from multiple user types. The fault sections database is projected in the WGS84 (EPSG:4326) coordinate reference frame.

### Database fields

The following section outlines and describes the fields included in the NSHM23 fault sections database (FSD):

1. FaultID: A unique fault ID is assigned to each fault following the convention of NSHM18. If a fault was previously included in prior releases of the NSHM, the fault ID number is consistent with prior use.
2. FaultName: A unique fault name is assigned. Faults with defined sections have additional section name information in parenthesis following the primary fault name.
3. PrimState: The primary state where the fault is located. If a fault crosses a state border, this is the state where most of the fault length resides.
4. SecState: If a fault crosses a state border, the secondary state is listed here. The minority of the fault length resides in this state, if listed.
5. DipDeg: The dip angle of a given fault, in degrees, from the surface of the earth onto the subsurface fault plane. Default value of 60° for reverse faults, 50° for normal faults, and 90° for strike-slip faults are used in absence of additional information.
6. DipDir: The cardinal orientation of primary dip direction. In the case of a 90° dipping fault, DipDir = “Vertical.”
7. Rake: The rake angle, in degrees, following standard conventions^40^.
8. LowDepth: The lower seismogenic depth (km; kilometers). A default value of 15 km is used in absence of additional information.
9. UpDepth: The upper seismogenic depth (km; kilometers). This value represents the depth of the buried fault trace in the case of blind faults. A default value of 0 km is used in absence of additional information.
10. Proxy: If this fault represents an extremely generalized view of distributed deformation and simplification of a polygon representing that distributed deformation zone, this value = “yes” and is otherwise left blank.
11. Linkto2014: If this fault was included in NSHM14/18, the ID number used in previous NSHM iterations is listed here. If a fault was not previously considered, the field is left blank.

The EQGeoDB is linked to the NSHM23 fault sections database via common values for FaultID and name. Because a single fault can have multiple entries in EQGeoDB, each entry (site) within EQGeoDB receives a unique identifier. The fields of NSHM23 EQGeoDB are:

1. SlipRateID: A unique site ID for each entry contained in the database.
2. FaultID: A unique fault ID is assigned to each fault following the convention of NSHM18. If a fault was previously included in prior releases of the NSHM, the fault ID number is consistent with prior use.
3. FaultName: A unique fault name is assigned. Faults with defined sections have additional section name information in parenthesis following the primary fault name.
4. State: Two-letter state abbreviation for site location.
5. SiteName: Name of site where slip rate observations and/or measurements were made, as reported by the original authors. If a categorical slip rate is applied, this field is labeled “approx centroid,” an abbreviation for approximate centroid.
6. DataType: Descriptor of slip rate source. If the slip rate is derived from a direct study, this field is filled with “slip rate study.” If a categorical rate is applied, the options for this field are: “QFFD slip rate bin.” Additional descriptors of rates include “dePolo and Anderson calibration rate,” “dePolo empirically-derived slip rate,” or “Utah hazard consensus rate.”
7. Observn: Observations used to derive slip rate as reported in original source.
8. PrefRate: Preferred rate if listed for a slip rate study. If this rate is reinterpreted from original source, details are provided in the column “ReptReint.” If rate is carried forward from UCERF3, Appendix B (field “AppB” = “yes”), this column contains the reported preferred geologic rate by the original authors as recorded in UCERF3 Appendix B.
9. LowRate: Lower boundary of rate. If DataType = “QFFD slip rate bin,” this is the lower bin of the QFFD range. If DataType = slip rate study, this is either the reinterpreted or reported lower boundary of the range of permissible slip rates (mm/yr; millimeters/year). If needed, calculation is explained in field “ReptReint.” If rate is carried forward from UCERF3, Appendix B (field “AppB” = “yes”), this column contains the reported preferred lower bound of geologic rate by the original authors.
10. HighRate: Upper boundary of rate. If DataType = “QFFD slip rate bin,” this is the upper bin of the QFFD range. If DataType = slip rate study, this is either the reinterpreted or reported upper boundary of the range of permissible slip rates (mm/yr; millimeters/year). If needed, calculation is explained in field “ReptReint.” If rate is carried forward from UCERF3, Appendix B (field “AppB” = “yes”), this column contains the reported preferred upper bound of geologic rate by the original authors.
11. RateUnct: Type of slip rate uncertainty. Values of uncertainty include 68% confidence interval (CI), 95% CI, and unknown. Any notes on uncertainty are included in parenthesis following a value.
12. RateType: Type of slip rate, describing what the slip rate is measuring and how it is applied to a given fault (e.g., “unprojected (vertical)”). No value is listed for QFFD slip rate bins.
13. ReptReint: Distinction between whether the rates reported here are as *reported*/defined by the original source, or if original data has been *reinterpreted* during compilation. If the latter, notes in this cell reflect how calculations are performed.
14. OffType: Basic description of offset measurement/feature.
15. AgeType: Basic description of age measurement/determination.
16. NumEvent: Number of reported surface-rupturing events at the site that comprise a given slip rate.
17. RateAge: Time interval over which rate is valid (kyr; thousands of years). Unless directly noted in this field, the slip rate is calculated between the present day and the age or age range listed in this field.
18. QbinMin: Lower boundary of QFFD slip rate bin (mm/yr; millimeters/year).
19. QbinMax: Upper boundary of QFFD slip rate bin (mm/yr; millimeters/year).
20. Reference: Reference(s) used to compile data for a given rate.
21. AppB: This column flags rates that have been carried over from UCERF3, Appendix B. If this field = “yes,” this rate was included in Appendix B.

## Technical Validation

**Fig. 9:**
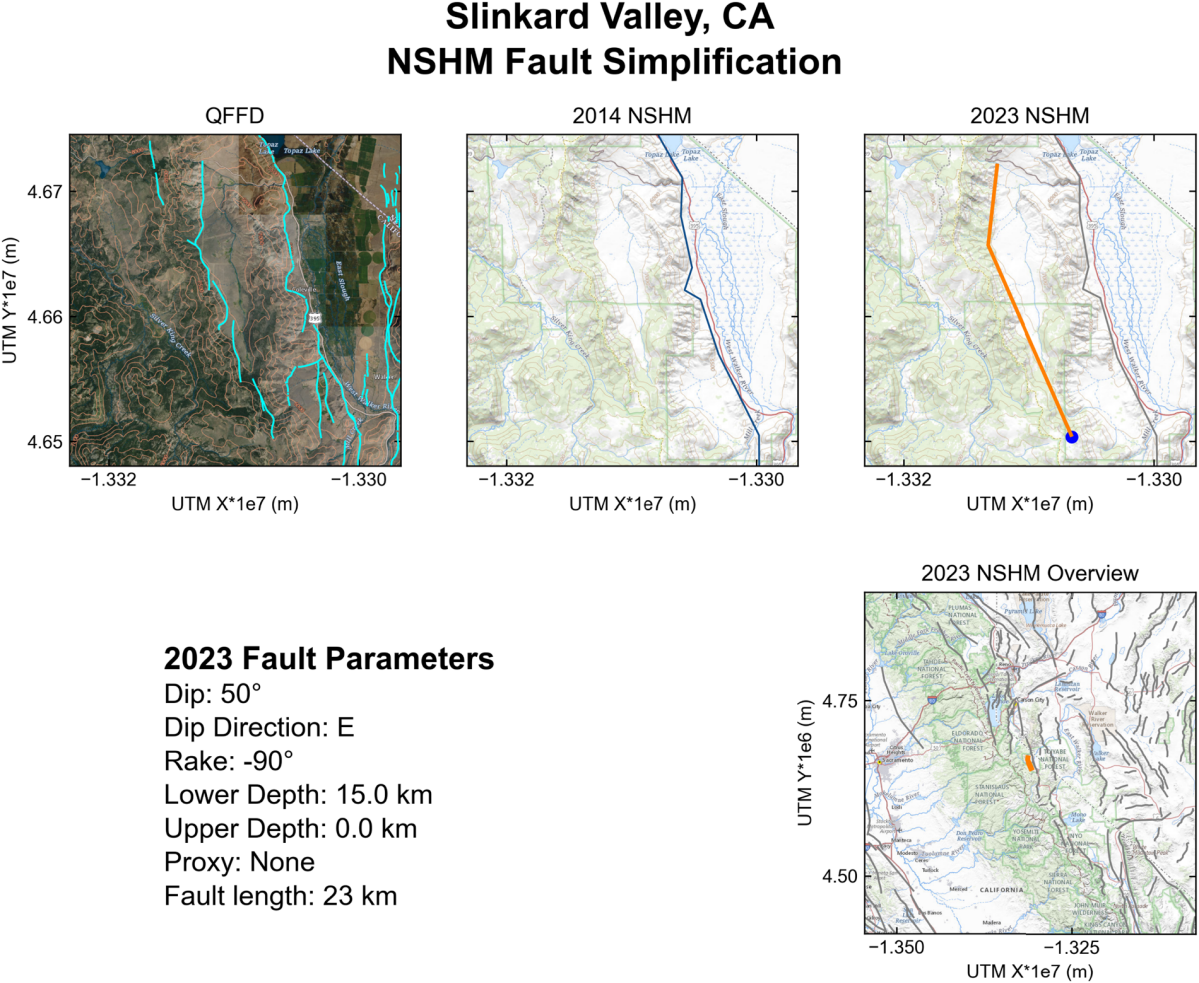
Example of output map page of Slinkard Valley fault (California), created using *nshm-faultmaps*^58^ during technical and scientific validation process. The top left panel shows the QFFD representation in the region in cyan; the top middle panel shows the lack of representation of the Slinkard Valley fault in NSHM14/18 FSD, which did include the nearby Antelope Valley fault (shown in blue); the top right panel shows the newly added representation of the Slinkard Valley fault in NSHM23 in orange. The blue dot at the southern extent of the NSHM23 fault trace shows the first node in the line geometry, indicating that this east-dipping fault abides by right-hand rule. The lower right panel shows a regional overview map, with Slinkard Valley fault highlighted in orange. Fault parameters from NSHM23 FSD are called and printed from the database in the text block at the lower left.

Database validation efforts focused on many numerical checks, including checking for duplicate database entries, draw direction/right-hand rule issues, multiple line segments comprising a single database entry, and fault naming/FaultID conventions. Manual visual review of each fault section was completed to further ensure that values such as dip degree, dip direction, and rake were tectonically consistent with the regional fault system and topography/geomorphology. To complete these visual reviews, we auto-generated maps for each fault section to visually confirm the validity of the geometry with respect to the local geology using the code *nshm-faultmaps*^58^. An example output from this code is shown from the Slinkard Valley fault of eastern California in Fig. 9. The example output highlights the QFFD mapping in the area, the lack of this fault in NSHM18 FSD, and the newly included NSHM23 fault section representation. Additionally, the page prints attributes from NSHM23 FSD. A user can use these codes to plot maps of all NSHM23 fault sections, or a particular region (e.g., State of Utah) or attribute (e.g., normal faults; rake = −90°). For more documentation on how to use and download this code, we encourage readers to visit the associated USGS data release at 10.5066/P9E3B8AG^58^.

Additional quality checks focused on a manual comparison of faults sections in the NSHM18 and NSHM23 FSDs. All fault attributes and node locations were compared. A detailed change log for each fault carried from NSHM18 to NSHM23 FSD is available^29,34^.

## Usage Notes

The NSHM23 FSD and EQGeoDB were intended for direct use in the 2023 release of the U.S. NSHM. Users interested in conducting other PSHA applications can ingest the fault sections database. We do not intend for this database to represent all observable faults at the surface. On the contrary, we intend for this database to represent simplified, idealized faults that extend to seismogenic depths. The EQGeoDB slip rate database can also be used as a guide for active tectonics researchers to plan field work, conduct systems-level research, and test hypotheses (e.g., regional comparison of geologic slip rates and geodetically constrained strain accumulation rates), and as input data/constraints in models (e.g., geodetic deformation models). We aim to augment the EQGeoDB with additional constraints on fault behavior, including paleoearthquake chronology and slip per event. The initial release of EQGeoDB contains only slip rates at points as this is the basic requirement for updating the NSHM23; future efforts may focus on the addition of paleoearthquake data and single-event displacements. We encourage readers to check the Community Page^57^ to find the most up-to-date version of the database, as updates to these databases may be periodically released.

The data can be viewed online by copying the geoJSON file into a free and open site such as geojson.io to quickly view the data. Additional mapping applications can ingest data as.shp format, such as ArcMap, QGIS, Google Earth Pro, or MATLAB. GeoJSON files are more widely readable by a large assortment of programs, including the above or other Python/Java libraries (e.g., OpenSHA).

An initial version of the databases (version 1) was released on January 21, 2021, which was published to begin deformation modeling work and preliminary implementation with the NSHM23 schedule. Version 1 has been superseded by version 2 (February 25, 2022) after numerical improvements to the representation of the database and additional data validation. We encourage users to refer to the Community Page^57^ to acquire any additional future updates to the databases.

## Data Availability

The code utilized to generate the individual fault maps in the visual verification and quality assurance of the database is written in Python 3.0 and is available at 10.5066/P9E3B8AG as a Jupyter Notebook^58^. This notebook is intended to share the plotting processes for how faults were visualized and can be manipulated by a user to prepare map images of specific faults or regions of choice.

## References

[1] Frankel A (1996). National Seismic-Hazard Maps: Documentation June 1996. U.S. Geol. Surv. Open-File Rep..

[2] Boncio P, Lavecchia G, Pace B (2004). Defining a model of 3D seismogenic sources for Seismic Hazard Assessment applications: The case of central Apennines (Italy). J. Seismol..

[3] Stirling M (2012). National seismic hazard model for New Zealand: 2010 update. Bull. Seismol. Soc. Am..

[4] Wesnousky SG (1986). Earthquakes, Quaternary Faults, and Seismic Hazard in California. J. Geophys. Res..

[5] Field EH, Jordan TH, Cornell CA (2003). OpenSHA: A developing community-modeling environment for seismic hazard analysis. Seismol. Res. Lett..

[6] Field, E. H. *et al*. Uniform California Earthquake Rupture Forecast, version 3 (UCERF3) -The time-independent model. *U.S. Geol. Surv. Open-File Rep*. 1122–1180 (2013).

[7] Petersen, M. D. *et al*. Geodesy- and geology-based slip-rate models for the Western United States (excluding California) national seismic hazard maps. *U.S. Geol. Surv. Open-File Rep*. 86 (2014).

[8] Nicol A, Van Dissen RJ, Stirling MW, Gerstenberger MC (2016). Completeness of the paleoseismic active-fault record in New Zealand. Seismol. Res. Lett..

[9] Page MT (2021). More fault connectivity is needed in seismic hazard analysis. Bull. Seismol. Soc. Am..

[10] Dawson, T. E. & Weldon, R. J. Appendix B — Geologic-Slip-Rate Data and Geologic Deformation Model. *Unif. Calif. Earthq. Rupture Forecast. Version 3 - Time-Independent Model* 1–29 (2013).

[11] Reitman NG, Molnar P (2021). Strain and Velocity Across the Great Basin Derived From 15-ka Fault Slip Rates: Implications for Continuous Deformation and Seismic Hazard in the Walker Lane, California-Nevada, USA. Tectonics.

[12] Bradley BA (2015). Benefits of site-specific hazard analyses for seismic design in New Zealand. Bull. New Zeal. Soc. Earthq. Eng..

[13] Petersen, M. D. *et al*. Documentation for the 2014 Update of the United States National Seismic Hazard Maps. *U.S. Geol. Surv. Open-File Rep*. 243 p. (2014).

[14] Earthquake Hazards Program, U. Quaternary Fault and Fold Database of the United States. https://www.usgs.gov/programs/earthquake-hazards/faults.

[15] Haller KM, Machette MN, Dart RL, Rhea BS (2004). U.S. Quaternary Fault and Fold Database Released. Eos (Washington. DC)..

[16] Schmitt, R. & Gold, R. D. Quaternary Fault and Fold Database of the United States. *U.S. Geological Survey Data Release* (2020).

[17] Dawson, T. E. Appendix A — Updates to the California Reference Fault Parameter Database — Uniform California Earthquake. *Unif. Calif. Earthq. Rupture Forecast. Version 3 - Time-Independent Model* 1–18 (2013).

[18] Faure Walker J (2021). Fault2SHA Central Apennines database and structuring active fault data for seismic hazard assessment. Sci. Data.

[19] Moschetti MP (2015). Seismic source characterization for the 2014 update of the U.S. National Seismic Hazard Model. Earthq. Spectra.

[20] Ben-Zion Y, Sammis CG (2003). Characterization of fault zones. Pure Appl. Geophys..

[21] Powers PM (2021). U.S. Geological Survey Software Release.

[22] Evans EL, Thatcher WR, Pollitz FF, Murray JR (2016). Persistent slip rate discrepancies in the eastern California (USA) shear zone. Geology.

[23] Evans EL (2018). A comprehensive analysis of geodetic slip-rate estimates and uncertainties in California. Bull. Seismol. Soc. Am..

[24] Evans EL, Loveless JP, Meade BJ (2015). Total variation regularization of geodetically and geologically constrained block models for the Western United States. Geophys. J. Int..

[25] Scharer, K. M. & Yule, D. A Maximum Rupture Model for the Southern San Andreas and San Jacinto Faults, California, Derived From Paleoseismic Earthquake Ages: Observations and Limitations. *Geophys. Res. Lett*. **47** (2020).

[26] Resor PG, Cooke ML, Marshall ST, Madden EH (2018). Influence of fault geometry on the spatial distribution of long-term slip with implications for determining representative fault-slip rates. Bull. Seismol. Soc. Am..

[27] Parsons, T. *et al*. Appendix C–Deformation Models for UCERF3. *U.S. Geol. Surv. Open-File Rep*. 1–66 (2013).

[28] Field EH, Milner KR, Page MT (2021). Generalizing the inversion-based PSHA source model for an interconnected fault system. Bull. Seismol. Soc. Am..

[29] Bender AM, Haeussler PJ, Powers PM (2021). U.S. Geological Survey.

[30] Bender AM, Haeussler PJ, Powers PM (2021). U.S. Geological Survey.

[31] Thompson Jobe JA (2022). U.S. Geological Survey.

[32] Angster SJ, Sherrod B, Bretthauer JK, Anderson ML (2020). U.S. Geological Survey.

[33] National Seismic Hazard Model Project, N. Request for Hazard Modeling Contributions | U.S. Geological Survey. https://www.usgs.gov/programs/earthquake-hazards/science/request-hazard-modeling-contributions?qt-science_center_objects=0.

[34] Hatem CM, Hatem AE, Briggs RB, Gold RD (2020). U.S. Geological Survey.

[35] Quaternary Fault and Fold Database of the United States. https://earthquake.usgs.gov/cfusion/qfault/query_main_AB.cfm?CFID=2273878&CFTOKEN=82f65fd1e3fe5cbe-187313DC-E19F-A27F-C62E4E84C438B037.

[36] Share PE (2020). Characterizing the uppermost 100 m structure of the San Jacinto fault zone southeast of Anza, California, through joint analysis of geological, topographic, seismic and resistivity data. Geophys. J. Int..

[37] Youngs RR (2003). A methodology for probabilistic fault displacement hazard analysis (PFDHA). Earthq. Spectra.

[38] Livio F, Serva L, Gürpinar A (2017). Locating distributed faulting: Contributions from InSAR imaging to Probabilistic Fault Displacement Hazard Analysis (PFDHA). Quat. Int..

[39] Baize S (2019). A worldwide and unified database of surface ruptures (SURE) for fault displacement hazard analyses. Seismol. Res. Lett..

[40] Aki, K. & Richards, P. G. *Quantitative seismology*. (University Science Books, 1980).

[41] Petersen, M. D. *et al*. Documentation for the 2008 update of the united states national seismic hazard maps. *Earthq. Res. Backgr. Sel. Reports* 107–234 (2010).

[42] Valentini A (2020). Relaxing segmentation on the Wasatch fault zone: Impact on seismic hazard. Bull. Seismol. Soc. Am..

[43] Friedrich AM, Wernicke BP, Niemi NA, Bennett RA, Davis JL (2003). Comparison of geodetic and geologic data from the Wasatch region, Utah, and implications for the spectral character of Earth deformation at periods of 10 to 10 million years. J. Geophys. Res. Solid Earth.

[44] Pérouse E, Wernicke BP (2017). Spatiotemporal evolution of fault slip rates in deforming continents: The case of the Great Basin region, northern Basin and Range province. Geosphere.

[45] Earthquake Hazards Program, U. 2014 National Seismic Hazard Maps - Source Parameters. https://earthquake.usgs.gov/cfusion/hazfaults_2014_search/query_main.cfm.

[46] dePolo CM, Anderson JG (2000). Estimating the slip rates of normal faults in the Great Basin, USA. Basin Res..

[47] dePolo, C. M. A Reconnaissance Technique for Estimating the Slip Rates of Normal-Slip Faults in the Great Basin and Application to Faults in Nevada, U.S.A. vol. 130 (University of Nevada, Reno, 1998).

[48] Lund, W. R. Consensus preferred recurrence-interval and vertical slip-rate estimates: Review of Utah Paleoseismic-Trenching Data by the by. *Utah Geol. Surv. Bull. 134* 1–114 (2005).

[49] Lund WR (2013). Working Group on Utah Earthquake Probabilies Preliminary Fault Characterization Parameters for Faults Common to the Working Group Study Areas and the U.S. National Seismic Hazard Maps. Utah Geol. Surv. Open-File Rep..

[50] Wong, I. *et al*. *Earthquake Probabilities for the Wasatch Front region in Utah, Idaho, and Wyoming*. *Utah Geological Survey Miscellaneous Publication 16-3* (2016).

[51] Styron R (2019). The impact of earthquake cycle variability on neotectonic and paleoseismic slip rate estimates. Solid Earth.

[52] Dieterich JH, Richards-Dinger KB (2010). Earthquake Recurrence in Simulated Fault Systems. Pure Appl. Geophys..

[53] Shaw BE (2018). A physics-based earthquake simulator replicates seismic hazard statistics across California. Sci. Adv..

[54] DuRoss CB (2020). Surface displacement distributions for the July 2019 Ridgecrest, California, earthquake ruptures. Bull. Seismol. Soc. Am..

[55] Thompson Jobe JA (2020). Evidence of previous faulting along the 2019 Ridgecrest, California, earthquake ruptures. Bull. Seismol. Soc. Am..

[56] Plesch A, Shaw JH, Ross ZE, Hauksson E (2020). Detailed 3D fault representations for the 2019 Ridgecrest, California, earthquake sequence. Bull. Seismol. Soc. Am..

[57] Hatem AE (2022). ScienceBase.

[58] Collett CM, Hatem AE, Reitman NG (2022). U.S. Geological Survey.

